# Inhibition of cortical synaptic transmission, behavioral nociceptive, and anxiodepressive-like responses by arecoline in adult mice

**DOI:** 10.1186/s13041-024-01106-5

**Published:** 2024-06-17

**Authors:** Qi-Yu Chen, Yuxiang Zhang, Yujie Ma, Min Zhuo

**Affiliations:** 1 School of Basic Medical Sciences, Fujian Medical University, Fuzhou, Fujian Province China; 2 CAS Key Laboratory of Brain Connectome and Manipulation, Interdisciplinary Center for Brain Information, Chinese Academy of Sciences Shenzhen Institute of Advanced Technology, Shenzhen, China; 3 Zhuomin International Institute for Brain Research, Qingdao, China; 4 Institute for Brain Research and Rehabilitation, South China Normal University, Guangzhou, China; 5 Oujiang Laboratory (Zhejiang Lab for Regenerative Medicine, Vision and Brain Health), Wenzhou, Zhejiang China; 6 Department of Physiology, Faculty of Medicine, University of Toronto, Medical Science Building, Room #3342, 1 King’s College Circle, Toronto, ON M5S 1A8 Canada

**Keywords:** Arecoline, Pain, Anxiety, Depression, Anterior cingulate cortex

## Abstract

Areca nut, the seed of Areca catechu L., is one of the most widely consumed addictive substances in the world after nicotine, ethanol, and caffeine. The major effective constituent of A. catechu, arecoline, has been reported to affect the central nervous system. Less is known if it may affect pain and its related emotional responses. In this study, we found that oral application of arecoline alleviated the inflammatory pain and its induced anxiolytic and anti-depressive-like behavior. Arecoline also increased the mechanical nociceptive threshold and alleviated depression-like behavior in naïve mice. In the anterior cingulate cortex (ACC), which acts as a hinge of nociception and its related anxiety and depression, by using the multi-electrode field potential recording and whole-cell patch-clamp recording, we found that the evoked postsynaptic transmission in the ACC of adult mice has been inhibited by the application of arecoline. The muscarinic receptor is the major receptor of the arecoline in the ACC. Our results suggest that arecoline alleviates pain, anxiety, and depression-like behavior in both physiological and pathological conditions, and this new mechanism may help to treat patients with chronic pain and its related anxiety and disorder in the future.

## Introduction

*Areca catechu L.* (Arecaceae) is the fourth most commonly used drug after tobacco, alcohol, and caffeine in the world [1]. It is consumed by approximately 10% of the world’s population, most of which are located in the tropical Pacific, Asia, and parts of East Africa. The users of the seeds of *Areca catechu L.*, areca nut, believe that it is helpful for the digestive system and has mild euphoric effects [2]. Arecoline (methyl-1, 2, 5, 6-tetrahydro-1-methyl-nicotinate), an alkaloid isolated from A. catechu, is considered the major effective constituent [3, 4]. Arecoline was reported to have wide pharmacological activities including effects on the nervous, cardiovascular, endocrine, and digestive systems. Arecoline could easily pass the blood-brain barrier (BBB) to activate the central muscarinic receptors [1, 5]. Furthermore, arecoline could be hydrolyzed into arecaidine, which was proved to be a potent inhibitor for uptake of the γ-aminobutyric acid (GABA) [6]. Therefore, the arecoline may have an inhibitory effect. Previous research demonstrated that a single intraperitoneal administration (i.p.) of arecoline could inhibit the locomotor activity in mice, and it also significantly shortened the duration of ethanol and phenobarbital sodium-induced loss of the righting reflex (LORR) [7, 8], indicating that arecoline had an excitatory effect. The other effect of arecoline is the enhancement of memory. Previous human and animal studies found that arecoline improved the ability of learning and memory in patients with Alzheimer’s Disease (AD) [9–11]. Additionally, in some early human studies, arecoline has also been used to induce rapid-eye movement (REM) sleep to study affective disorders such as anxiety and depression [12–14]. Although there are some reports that arecoline has analgesic and anxiolytic effects [15–18], few reports investigated the role of arecoline in chronic pain and its accompanying anxiety and depression.

Chronic pain and its related anxiety and depression are always encountered in clinical situations. Preclinical animal studies also reported depression- and anxiety-like behaviors in different chronic pain models [19]. In cerebral cortices, the anterior cingulate cortex (ACC) plays a pivotal role in modulating chronic pain and its related anxiodepressive-like behavior [20–23]. It was reported that the long-term enhancement of excitatory transmission in the ACC promoted chronic pain and its related anxiety and depression. Drugs that target the excitatory transmission in the ACC may have potential in the treatment of chronic pain and its related anxiodepressive emotions.

In the current study, we investigated the role of arecoline in chronic pain and its related anxiety and depression. We found that the arecoline alleviated the mechanical allodynia and anxiodepressive behavior by inhibiting the evoked postsynaptic transmission in the ACC, which becomes a possible effect of areca nuts/betel chewing.

## Materials and methods

### Animals

Adult male and female C57BL/6 mice (7–9 weeks old) were used. All animals were housed under a 12 h light/dark cycle with food and water provided *ad libitum*. Experiments were conducted under the protocol approved by the Animal Care and Use Committees at the Oujiang Laboratory (OJLAB22121612).

### Animal models

For the Complete Freund’s adjuvant (CFA) model, 50% CFA (10 µl; Sigma-Aldrich) was injected subcutaneously into the dorsal side of the left hind paw as reported previously [24]. For the arecoline-drinking mice, 50 mL 0.2% arecoline hydrobromide salt was dissolved in sterile tap water and provided ad libitum for two weeks [25, 26]. Water was provided in the control group.

### Behavioral test

#### Tail-flick reflex

The spinal nociceptive tail-flick reflex was evoked by focused, radiant heat (Columbus Instruments) provided by a 50-W projector lamp focused on a 1.5-mm by 10-mm area on the underside of the tail. The latency to reflexive removal of the tail from the heat was measured by a digital photocell timer to the nearest 0.1 s. The cutoff time of 10 s was used to minimize damage to the skin of the tail. All behavioral tests were performed at 10-min intervals. The response latency was an average of three or four measurements.

#### Hot-plate test

The hot plate consisted of a thermally controlled 25.4-cm by 25.4-cm metal plate (55 °C) surrounded by four Plexiglas walls (Columbus Instruments). The time between the placement of the mouse on the plate and the licking or lifting of a hind paw or jumping was measured with a digital timer. Mice were removed from the hot plate immediately after the first response. The cutoff time of 30 s was imposed to prevent tissue damage. All behavioral tests were performed at 10-min intervals. The response latency was an average of three or four measurements.

#### Mechanical allodynia

Mice were individually placed in a round, transparent container 20 cm in diameter and were allowed to acclimate for 30 min before testing. The mechanical threshold was assessed based on the responsiveness of the hind paw to the application of von Frey filaments (Ugo Baslie) to the point of bending. The filament was applied over the dorsum of the paw while the animal was resting. Positive responses included licking, biting, and sudden withdrawal of the hindpaw.

#### Elevated plus maze

The elevated plus maze (VanBi Tec.) consisted of two open arms and two closed arms situated perpendicular to each other. The maze was situated ~ 70 cm from the floor. For each test, mice were individually placed in the center square and allowed to move freely for 5 min. The number of entries and time spent in each arm were recorded. A video camera tracking system was used to generate the traces.

#### Open-field test

To record locomotor activity, we used an open-field activity monitor (40 cm by 40 cm by 30.5 cm; VanBi Tec.). Briefly, this system uses paired sets of photo beams to detect movement in the open field, and movement is recorded as beam breaks. The open field is placed inside an isolation chamber with dim illumination. Each subject was placed in the center of the open field. Locomotor activity was then measured for 30 min.

#### Forced swimming test

Mice were into a glass cylinder (height 17.5 cm, diameter 12.5 cm) containing 11.5 cm of water (23–25 °C). The test duration was 6 min; but because only little immobility was observed during the first 2 min, we only quantified the duration of immobility during the last 4 min of the test. Mice were considered to be immobile when they floated upright in the water, with only minor movements to keep their heads above the water.

#### Tail suspension test

The apparatus for the tail suspension test (TST) was a white plexiglass box with a hook on the ceiling of the box. The mice were suspended 15 cm above the floor on the hook with tape at 2 cm proximal to the tail tip. The test session lasted 6 min and was recorded with a video camera. The freezing time was scored for the last 4 min of the session.

### Drugs

The chemicals and drugs used in this study were as follows: Picrotoxin, tetrodotoxin, and CNQX were purchased from HelloBio (Princeton, NJ, USA), arecoline was purchased from Absin (Shanghai, China), carbachol was bought from Topscience biochem (Shanghai, China). Drugs were prepared as stock solutions for frozen aliquots at -20℃. All drugs were diluted from the stock solution to the final desired concentration in the ACSF before being applied to the perfusion solution.

### Brain slices preparation

Coronal brain slices (300 μm) of ACC were prepared using standard methods. Briefly, mice were deeply anesthetized with 5% isoflurane and sacrificed by decapitation. The whole brain was removed quickly from the skull and submerged in the oxygenated (95% O_2_ and 5% CO_2_) ice-cold artificial cerebrospinal fluid (ACSF) containing (in mM) 124 NaCl, 2.5 KCl, 2 MgSO_4_, 1 NaH_2_PO_4_, 2 CaCl_2_, 25 NaHCO_3_, and 10 D-glucose. The whole brain tissue was cooled for a short time before the tissue containing the target region was isolated and glued onto the vibratome (VT1200S Vibratome, Leica, Germany). Slices were incubated in a submerged recovery chamber at room temperature for one hour. The ACSF was continuously balanced with a mixture of 95% O_2_ and 5% CO_2_.

### Multi-channel field potential recordings

The procedures for the preparation of the MED64 were similar to that of the previous report [27]. The MED64 probe (MED-P515A, 8 × 8 array, interpolar distance 150 μm, Panasonic) was superfused with normal ACSF (pH = 7.4) at 28 –30 °C and maintained at a 2 ml/min flow rate. Slices were kept in the recording chamber for at least 1 h before the start of experiments. One planar microelectrode with bipolar constant current pulses (10–100 µA, 0.2 ms) was used for stimulation of the ACC slice. The channels with fEPSP were considered as active channels and their fEPSP responses were sampled every 1 min and averaged every 4 min. The parameter of ‘slope’ indicated the average slope of each fEPSP recorded by activated channels.

### Whole-cell patch-clamp recording

Whole-cell recordings were performed in a recording chamber on the stage of an Olympus BX51 microscope with infrared differential interference contrast (DIC) optics for visualization. EPSCs were recorded from layer II/III neurons with a HEKA amplifier (EPC 10, HEKA), and the stimulations were evoked in layer V of the ACC by a bipolar tungsten stimulating electrode. When the EPSCs were recording, the recording pipettes (3–5 MΩ) were filled with the solution containing (in mM) 145 K-gluconate, 5 NaCl, 1 MgCl_2_, 0.2 EGTA, 10 HEPES, 2 Mg-ATP, and 0.1 Na_3_-GTP, which adjusted to pH 7.3 with KOH and had osmolality of 290 mOsmol. Picrotoxin (100 µM) was always present to block GABA_A_ receptor-mediated inhibitory synaptic currents in all the EPSC recordings. The amplitudes of evoked EPSCs were adjusted to between 100 and 150 pA to obtain a baseline. IPSCs were recorded with bath presence of CNQX (20 mM) and holding 0 mV. The patch electrode internal solution (in mM) 112 Cs-Gluconate, 5 TEA-Cl, 3.7 NaCl, 0.2 EGTA, 10 HEPES, 2 Mg-ATP, 0.1 Na_3_-GTP, and 5 QX-314 (adjusted to pH 7.2 with CsOH, 290 mOsmol) were used for recording IPSCs. For miniature EPSCs/IPSCs (mEPSCs/mIPSCs) recordings, tetrodotoxin (TTX, 1 µM) was added to the perfusion solution. The evoked E-I ratio was defined with the ratio of the amplitude of eEPSCs and eIPSCs when the neurons were held in the − 60 mV and 0 mV respectively. Access resistance was 15–30 MΩ and monitored throughout the experiment. Data was collected only when access resistance changed < 15% during all experiments. Data was filtered at 1 kHz and digitized at 10 kHz.

### Statistical analysis

GraphPad Prism 8 (GraphPad Software Inc., San Diego, CA, USA) was used for plotting figures, and SPSS version 22.0 (SAS Institute Inc, Cary, NC) software was used to analyze the results. The paired t-tests or two-way ANOVA were conducted as appropriate. All data were presented as the mean ± standard error of the mean (SEM). In all cases, *p* < 0.05 was considered statistically significant.

## Results

### Drinking arecoline alleviates the allodynia caused by CFA injection

**Fig. 1:**
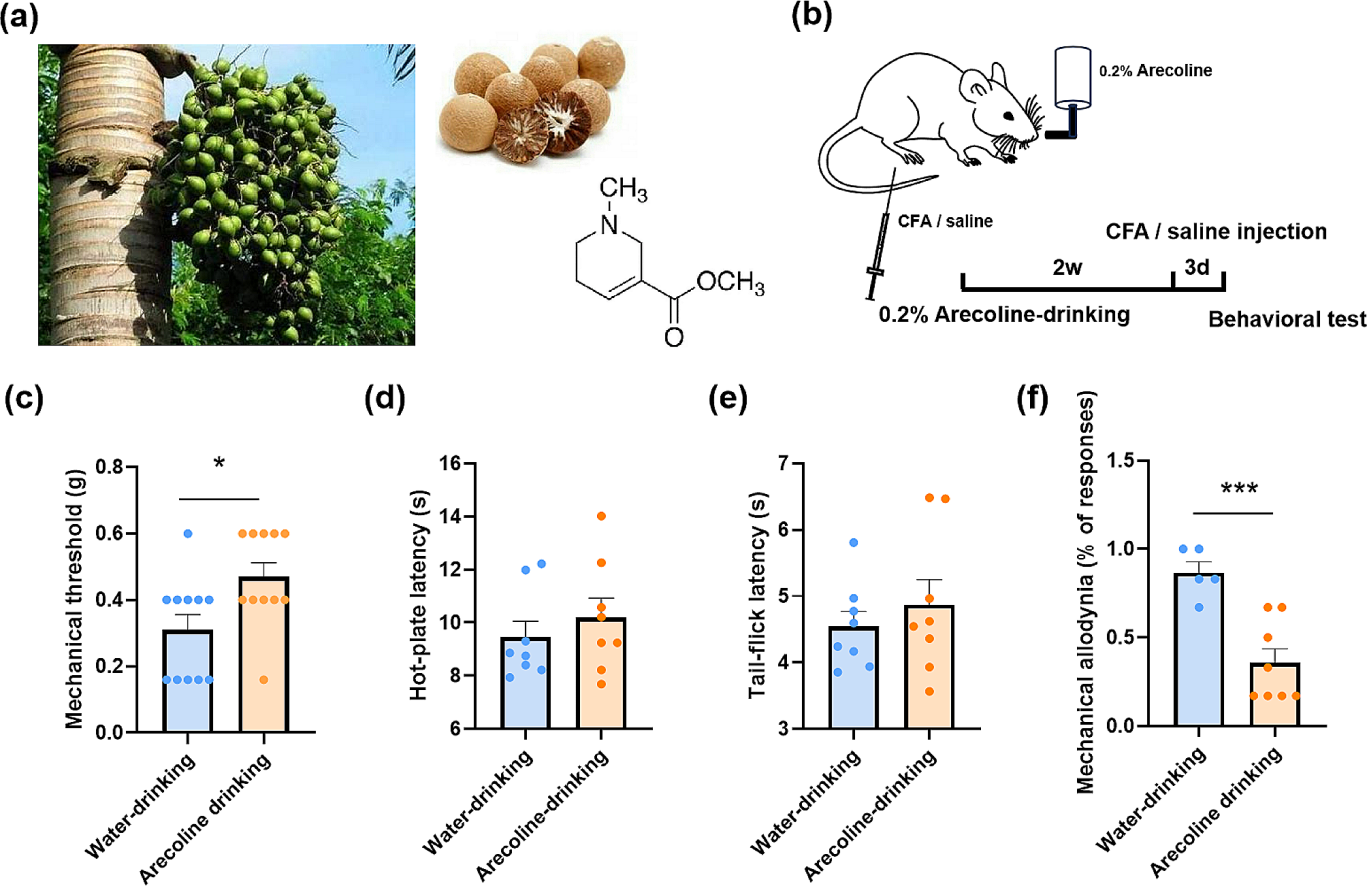
The analgesic effect of drinking arecoline on acute and inflammatory pain in mice. (**a**) Photographs of the fresh and dry betel nuts and the structural formula of the arecoline. (**b**) Schematic showing the arecoline (0.2%) was added into the water bottle of the mice and let them drink for 2 weeks. CFA was injected three days before the behavioral test. (**c**) The mechanical threshold of the naive mice drinking water (blue) or arecoline (orange). *p < 0.05, compared to the water-drinking group. *n* = 11 for arecoline-drinking group, *n* = 11 for the water-drinking group. (**d**) Latency on the 55℃ hot plate of naive mice drinking water or arecoline. *n* = 8 for arecoline-drinking group, *n* = 8 for the water-drinking group. (**e**) Tail-flick latency of naive mice drinking water or arecoline. (**f**) The mechanical allodynia of the CFA model drinking water or arecoline. ***p < 0.001, compared to the water-drinking group. *n* = 8 for arecoline-drinking group, *n* = 5 for the water-drinking group

The arecoline is subtracted from the areca nuts (Fig. 1a). To investigate the effect of arecoline on pain behavior, we added the arecoline (0.2%) into the drinking water of the mice and let them drink *ad libtum* for 2 weeks (Fig. 1b). Drinking arecoline increased the mechanical nociceptive threshold (Water-drinking: 0.31 ± 0.05 g vs. arecoline-drinking 0.47 ± 0.04 g, unpaired t-test, t_(20)_ = 2.5, *p* = 0.02, *n* = 11 for arecoline-drinking group, *n* = 11 for the water-drinking group, Fig. 1c) without affecting the thermal pain (For the hot-plate test, water-drinking 9.5 ± 0.6 s vs. arecoline-drinking 10.2 ± 0.8 s, unpaired *t*-test, t_(14)_ = 0.76, *p* = 0.46, *n* = 8 for arecoline-drinking group, *n* = 8 for the water-drinking group, Fig. 1d; For the tail-flick test, water-drinking: 4.5 ± 0.2 s vs. arecoline-drinking 4.9 ± 0.4 s, unpaired *t*-test, t_(14)_ = 0.73, *p* = 0.48, *n* = 8 for arecoline-drinking group, *n* = 8 for the water-drinking group, Fig. 1e). On the 3rd day after the intraplantar injection of CFA, the allodynia was alleviated (water-drinking 0.87 ± 0.05 vs. arecoline-drinking 0.36 ± 0.09, unpaired *t*-test, t_(11)_ = 4.5, *p* = 0.0009, *n* = 8 for arecoline-drinking group, *n* = 5 for the water-drinking group, Fig. 1f) These results indicate that the arecoline reversed the mechanical hypersensitization in mice.

### Arecoline alleviates the anxiety and depression induced by CFA injection

**Fig. 2:**
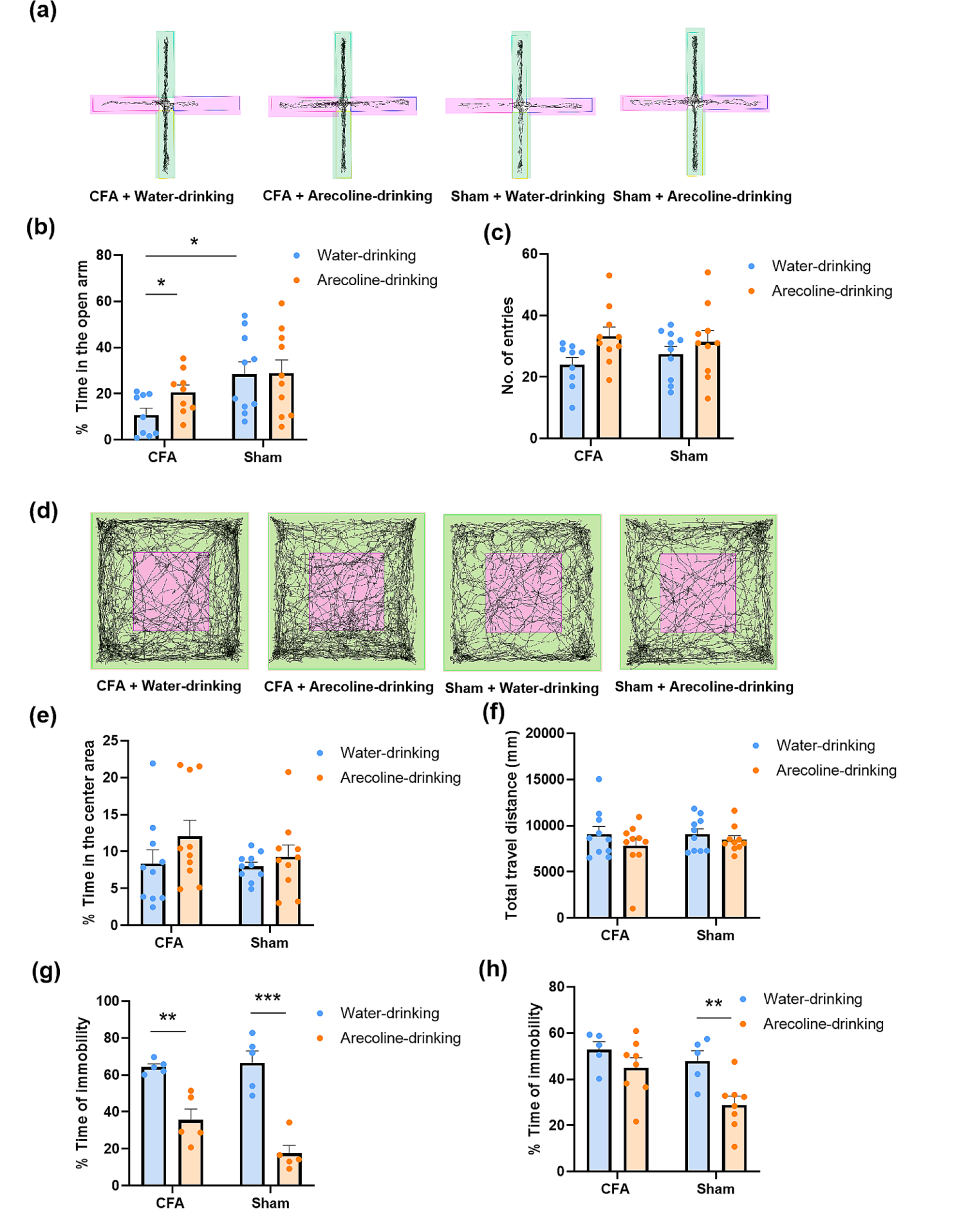
The anxiolytic and anti-depressive effect of drinking arecoline in the CFA-injected mice. (**a**) Representative traces of mice in CFA or sham group drinking water or arecoline in the EPM (open arms in pink, closed arms in green). (**b**) The percentage of time in the open arms for CFA + water-drinking, CFA + arecoline-drinking group, sham + water-drinking group, and sham + arecoline-drinking group. *n* = 9 for the CFA group, *n* = 10 for the sham group, *p < 0.05, compared to the water-drinking mice in the CFA group. (**c**) The number of total entries in the EPM for the four groups. (**d**) Representative traces of mice in CFA or sham group drinking water or arecoline in the OF (center area in pink, peripheral area in green). (**e**) The percentage of time in the center area of the OF for the four groups. *n* = 10 for CFA group, *n* = 10 for the sham group. (**f**) The number of total travel distances in the OF for the four groups. (**g**) The percentage of time of immobility in the FST for the four groups. *n* = 5 for CFA group, *n* = 5 for the sham group. **p < 0.01, ***p < 0.001, compared to water-drinking group. (h)The percentage of time of immobility in the TST for the four groups. *n* = 5 for CFA group, *n* = 5 for the sham group. **p < 0.01, compared to water-drinking group

To further investigate the effect of the arecoline on the emotional component of the CFA-induced pain, we measured the anxiodepressive behavior after the animals drank the 0.2% arecoline for 2 weeks. The CFA-injected mice showed decreased exploration in the open arms compared to the sham group (for the water-drinking group, CFA 10.8 ± 2.9% vs. Sham 28.4 ± 5.4%, two-way ANOVA, F _(1, 34)_ = 7.752, Sidak’s multiple comparisons test, *p* = 0.02, *n* = 9 for CFA group, *n* = 10 for the sham group). Arecoline reversed this anxiety-like phenomenon (for the arecoline-drinking group, CFA 20.6 ± 3.1% vs. Sham 28.9 ± 5.8%, Sidak’s multiple comparisons test, *p* = 0.40, *n* = 9 for CFA group, *n* = 10 for the sham group, Fig. 2a to c). However, in the open-field test, arecoline did not show a significant difference in the time in the center area (for the water-drinking group, CFA 8.4 ± 1.9% vs. Sham 8.0 ± 0.6%, two-way ANOVA, F _(1, 36)_ = 0.94, Sidak’s multiple comparisons test, *p* = 0.98, *n* = 10 for CFA group, *n* = 10 for the sham group; for the arecoline-drinking group, CFA 12.1 ± 2.1% vs. Sham 9.3 ± 1.6%, Sidak’s multiple comparisons test, *p* = 0.42, *n* = 10 for CFA group, *n* = 10 for the sham group, Fig. 2d to f). These data indicate that the arecoline has an anxiolytic effect on the CFA mice.

To observe the effect of arecoline on depression-like behavior, we did the forced-swim test (FST) and the tail-suspension test (TST). Arecoline decreased the immobility time in both the CFA and sham mice in the FST (for the CFA group, water-drinking 64.4 ± 1.6% vs. arecoline-drinking 35.6 ± 5.9%; for the sham group, water-drinking 66.6 ± 6.5% vs. arecoline-drinking 17.5 ± 4.4%, two-way ANOVA, F _(1, 16)_ = 61.06, in CFA group, Sidak’s multiple comparisons test, *p* = 0.0017, *n* = 5 for the water-drinking group, *n* = 5 for the arecoline-drinking group; in the sham group, Sidak’s multiple comparisons test, *p* < 0.0001, *n* = 5 for water-drinking group, *n* = 5 for the arecoline-drinking group, Fig. 2g). In the TST, only mice in the sham group showed decreased immobility after drinking the arecoline (for the CFA group, water-drinking 52.8 ± 3.5% vs. arecoline-drinking 45.0 ± 4.4%; for the sham group, water-drinking 47.9 ± 4.4% vs. arecoline-drinking 28.8 ± 3.8%, two-way ANOVA, F _(1, 22)_ = 9.70, in CFA group, Sidak’s multiple comparisons test, *p* = 0.38, *n* = 5 for the water-drinking group, *n* = 8 for the arecoline-drinking group; in sham group, Sidak’s multiple comparisons test, *p* = 0.0098, *n* = 5 for water-drinking group, *n* = 8 for the arecoline-drinking group, Fig. 2h). These data indicate that arecoline causes anti-depressive effects.

### Arecoline inhibits the excitatory field potentials in the anterior cingulate cortex

**Fig. 3:**
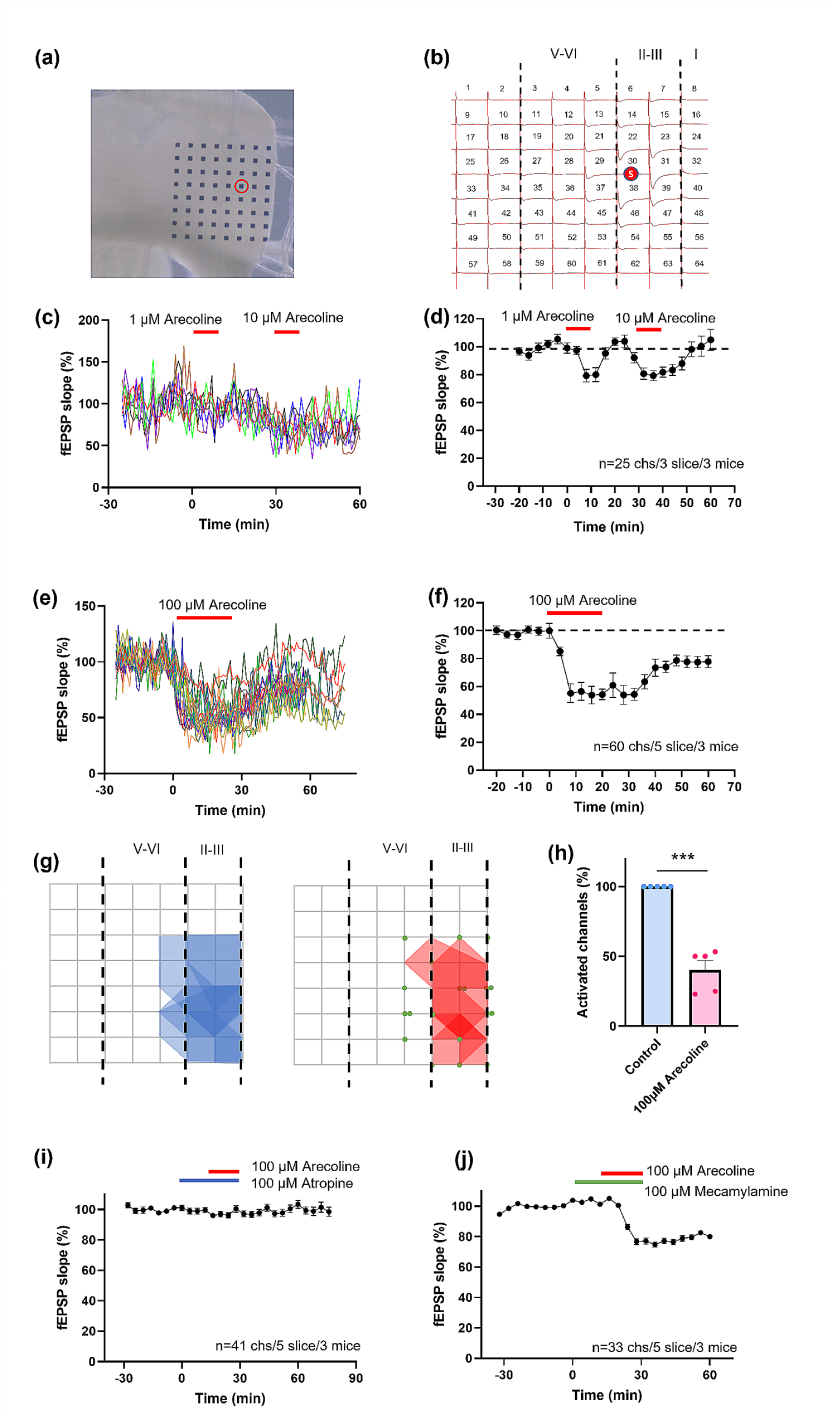
The inhibition effect of arecoline on the excitatory field potentials in the ACC. (**a**) Microphotograph showed one example of ACC fEPSP recording by using the MED64 system. A cortex slice containing the ACC was placed on a probe with 64 electrodes (MED-P515A, 8 × 8 array). One channel of the probe (red circle) was selected as the stimulation site. (**b**) mapped figure showing the evoked potentials from all channels induced by one stimulation channel in the superficial layers (layer II/III) of the ACC. (**c**) The fEPSP slope of 7 channels from one slice of the ACC before, during, and after the application of 1 µM and 10 µM arecoline. The red line indicates the time when the arecoline was perfused. (**d**) Time course of the averaged fEPSP slope of 25 recorded channels in 3 slices/ 3 mice before, during, and after the application of 1 µM and 10 µM arecoline. (**e**) The fEPSP slope of 16 channels before, during, and after the application of 100 µM arecoline. The red line indicates the time when the arecoline was perfused. (**f**) Time course of the averaged fEPSP slope of 60 recorded channels in 5 slices/ 3 mice before, during, and after the application of 100 µM arecoline. (**g**) The polygonal diagram shows the baseline area of the activated channels with fEPSP (blue) and the decreased area after 100 µM arecoline application (red). The silenced channels are shown as green dots. In some sites, more than one green dot is shown. It means the silenced fEPSPs could be observed in the same channel in different slices. (**h**) Bar histogram of grouped data (*n* = 5 slices/3 mice) showing the percentage of silenced channels in the superficial layer of the ACC. (**i**) The averaged fEPSP slope of 41 recorded channels in 5 slices/ 3 mice before, during, and after the application of 100 µM atropine and arecoline. The arecoline (short red line) was perfused 15 min after the 100 µM atropine (short blue line) was perfused. (**j**) The averaged fEPSP slope of 33 recorded channels in 5 slices/ 3 mice before, during, and after the application of 100 µM mecamylamine and arecoline. The arecoline (short red line) was perfused 15 min after the 100 µM mecamylamine (short green line) was perfused

Since the ACC plays an important role in pain-related anxiety, we observed the effect of arecoline on the coronal slices of the ACC by using the 64-electrode recording system. As shown in Fig. 3a and b, one channel in the superficial layer of the ACC (layer II/III) was chosen as the stimulation site, and the other 63 channels were used for measuring evoked responses. We found that local stimulation induced widespread fEPSPs within the ACC. After bath application of the arecoline (1 µM), the fEPSPs were temporarily inhibited (80.0 ± 5.0% of baseline, 25 channels/3 slice/3 mice, Fig. 3c and d). After increasing the dose of arecoline to 10 µM and 100 µM, the duration and the percentage of inhibition increased (10 µM arecoline: 79.3 ± 3.6% of baseline, *n* = 25 channels /3 slice/3 mice; 100 µM arecoline: 53.8 ± 6.3% of baseline, *n* = 60 channels /5 slice/3 mice, Fig. 3c to f). Application of 100 µM arecoline decreased the number of active channels (40.3 ± 6.7% of baseline, *n* = 5 slice/3 mice, Fig. 3g, 3 h). According to previous studies, the arecoline is an agonist of cholinergic receptors. Therefore, by applying the selective antagonists of muscarinic and nicotinic receptors, we investigated their effect on the inhibition effect of arecoline. After perfusing the antagonist of muscarinic receptor atropine (100 µM), the inhibition effect of the arecoline was blocked (100.4 ± 2.2% of baseline, *n* = 41 channels /5 slice/3 mice, Fig. 3i). However, the antagonist of nicotinic receptor mecamylamine (100 µM) did not block the inhibition of arecoline (74.8 ± 1.7% of baseline, *n* = 33 channels /5 slice/3 mice, Fig. 3j). These data indicate that in the ACC, arecoline caused an inhibitory effect on excitatory transmission mainly via muscarinic receptors.

### The inhibition effect of arecoline in the ACC is postsynaptic

**Fig. 4:**
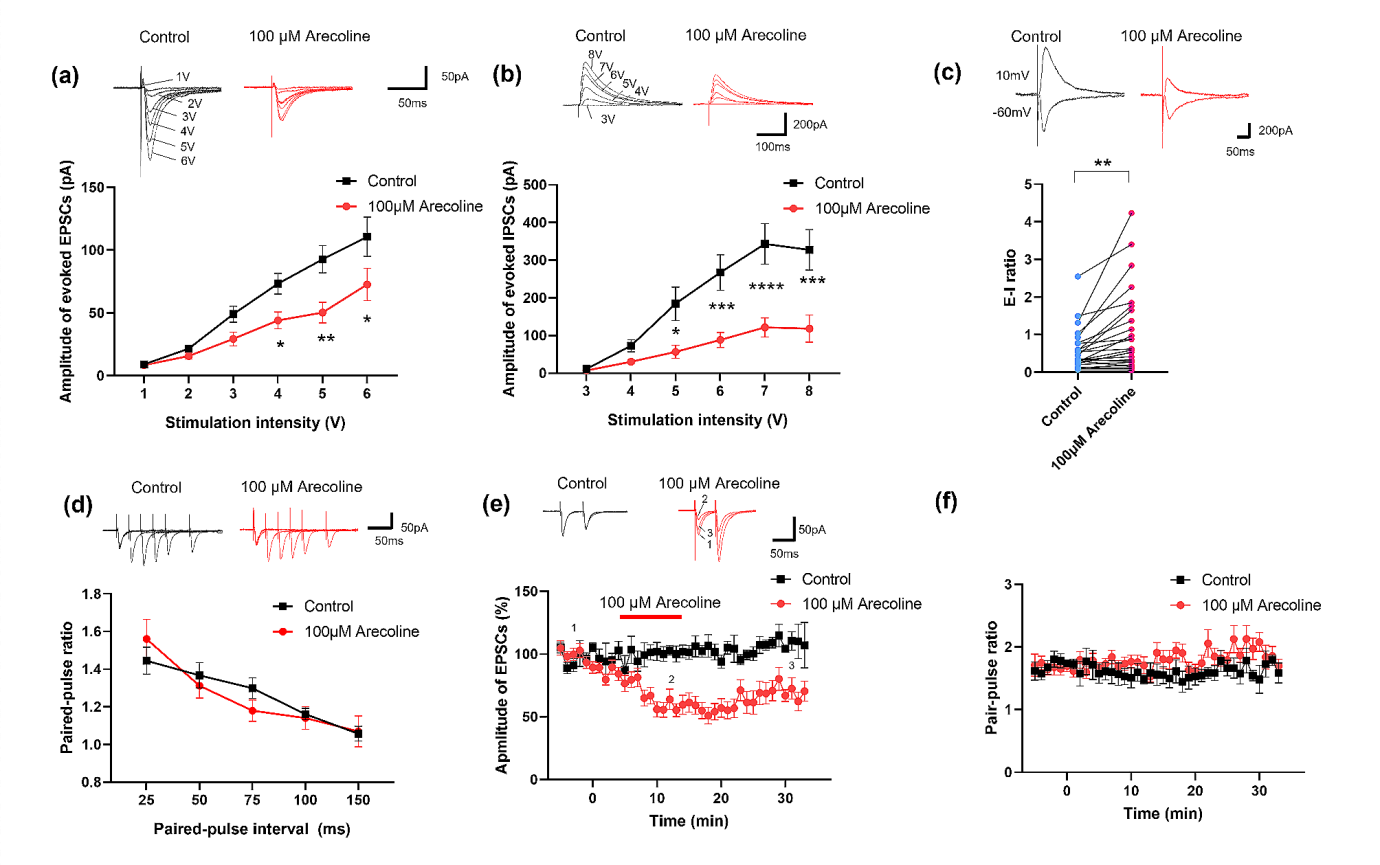
Decreased evoked synaptic transmission in layer II/III of the ACC after the application of arecoline (**a**) Representative traces (top) and input-output curves (bottom) of evoked EPSC in ACC slices before and after the arecoline application. *n* = 9 neurons/6 mice. *p < 0.05 and **p < 0.01 compared with baseline control. (**b**) Representative traces (top) and input-output curves (bottom) of evoked IPSC in ACC slices before and after the arecoline application. *n* = 10 neurons/5 mice. *p < 0.05 and ***p < 0.001, ****p < 0.0001 compared with baseline control. (**c**) Top: representative traces of evoked excitatory and inhibitory responses recorded before and after the application of arecoline in the ACC. Bottom: the evoked E-I ratio before and after the application of arecoline. *n* = 25 neurons/11 mice. (**d**) Representative traces (top) and PPR before and after the arecoline application. *n* = 9 neurons/6 mice. (**e**) Representative traces (top) and plot of the time course of arecoline inhibiting the amplitude of paired pulse-induced EPSCs (bottom). The red line indicates the time when the arecoline was perfused. (**f**) Time course of the PPR before, during, and after the application of arecoline. *n* = 14 cells/ 7 mice

To further investigate the effect of arecoline on the neurons in the ACC, we performed whole-cell patch-clamp recording, we recorded the excitatory postsynaptic currents (EPSCs) and the inhibitory postsynaptic currents (IPSCs) in pyramidal neurons in the layer II/III of the ACC in mice. We found that the input (stimulation intensity)-output (EPSC or IPSC amplitude) curve was significantly shifted to the right after applying the arecoline (100 µM) compared with that in control group (*n* = 9 neurons/6 mice, two-way ANOVA, F _(1, 81)_ = 22.5, *p* < 0.0001, Fig. 4a; *n* = 10 neurons/5 mice, two-way ANOVA, F _(1, 102)_ = 50.7, *p* < 0.0001, Fig. 4b). We then investigate whether the arecoline changes the ratio of excitatory/inhibitory (E/I ratio). The evoked EPSCs were recorded with the holding potential of -60 mV and evoked IPSCs were recorded with the holding potential of 0 mV. We found that both the excitatory and inhibitory evoked responses were inhibited after the application of arecoline, while the evoked E-I ratio increased after applying the arecoline in comparison with baseline in the ACC (control 0.6 ± 0.1 vs. arecoline: 1.1 ± 0.2, paired t-test, t _(24)_ = 3.598, *p* = 0.0014, *n* = 25 cells/ 11 mice, Fig. 4c). These data indicate that the arecoline inhibits the synaptic transmission in the ACC, and the inhibitory effect on the inhibitory synaptic transmission seems stronger.

To examine whether presynaptic or postsynaptic mechanisms mediate the decreased excitatory synaptic transmission in the ACC, we examined the paired-pulse ratio (PPR) was examined in ACC neurons. PPR was observed at different stimulus intervals of 35, 50, 75, 100, and 150 ms. After the application of arecoline, there was no significant change in PPR in ACC neurons compared with baseline (*n* = 9 neurons/6 mice, two-way ANOVA, F _(1, 75)_ = 0.45, *p* = 0.50, Fig. 4d). We also recorded the PPR with fixed interval (50 ms) before and during the application of arecoline. We found that the amplitude of EPSC decreased to 50.9 ± 6.7% of baseline (*n* = 10 cells/4 mice, Fig. 4e), while the PPR had no significant change (Fig. 4f). These results suggest that the arecoline mainly inhibits the postsynaptic transmission in the ACC neurons.

**Fig. 5:**
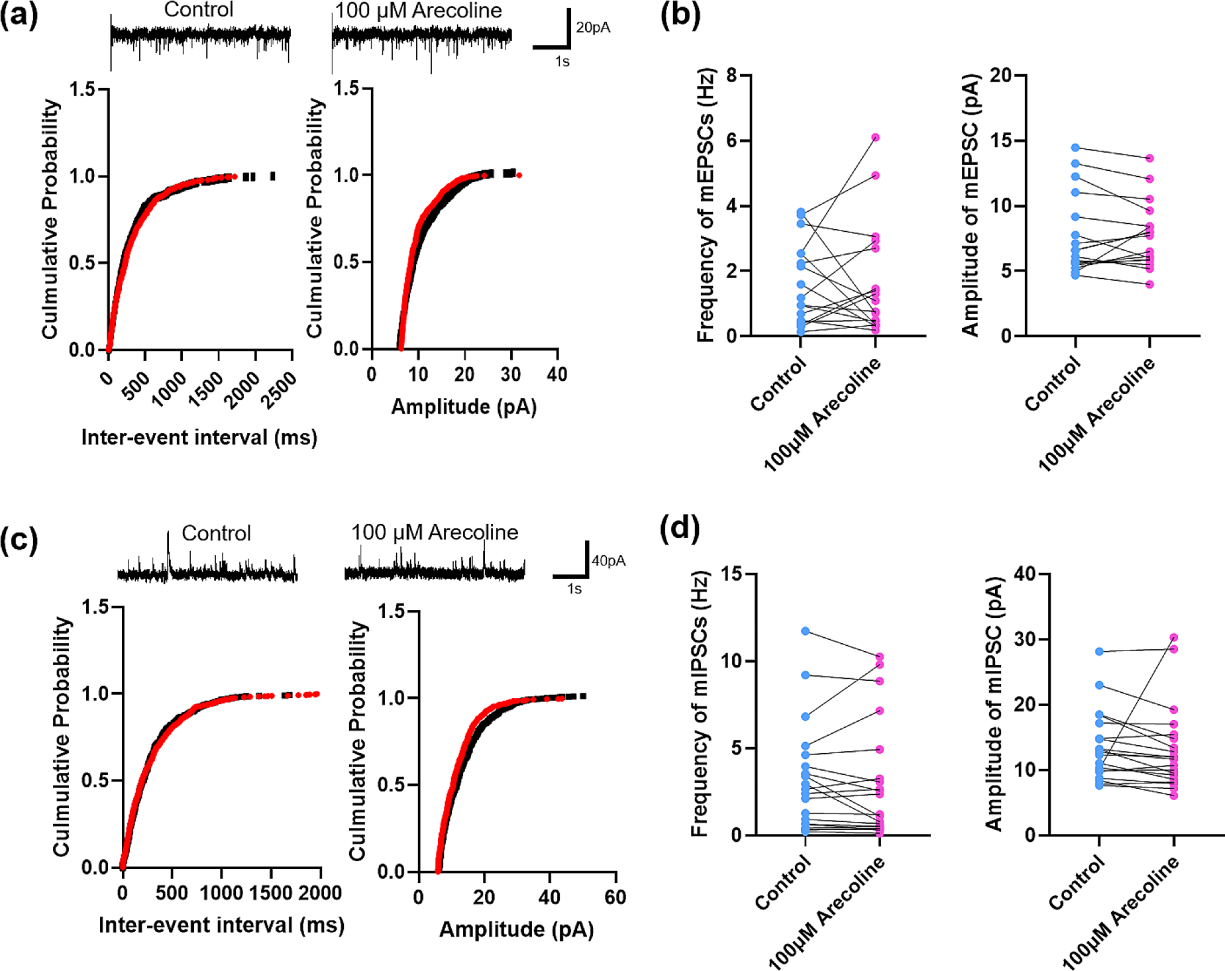
Changes of mEPSCs and mIPSCs in layer II/III of the ACC after the application of arecoline (**a**) Representative mEPSCs recorded in the ACC neuron in slices before (upper left) and after (upper right) the arecoline perfusion. Cumulative interevent interval (lower left) and amplitude (lower right) histograms of mEPSCs before (black square) and after (red circle) the arecoline perfusion. (**b**) Summary plots of mEPSC data. Averaged values of mEPSC parameters: mean peak frequency (left) and amplitude (right). *n* = 18 cells/ 7 mice (**c**) Representative mIPSCs recorded in the ACC neuron in slices before (upper left) and after (upper right) the arecoline perfusion. Cumulative interevent interval (lower left) and amplitude (lower right) histograms of mIPSCs before (black square) and after (red circle) the arecoline perfusion. (**d**) Summary plots of mIPSC data. Averaged values of mIPSC parameters: mean peak frequency (left) and amplitude (right). *n* = 20 cells/ 7 mice

We further investigated the effect of arecoline on spontaneous synaptic transmission, we recorded the miniature EPSCs and IPSCs (mEPSCs and mIPSCs) in the ACC. However, we found neither the frequency nor the amplitude of the mEPSC changed after the arecoline perfusion (frequency: control 1.6 ± 0.3 Hz vs. arecoline 1.7 ± 0.4 Hz, paired t-test, t _(17)_ = 0.17, *p* = 0.87; amplitude: control 7.8 ± 0.8 pA vs. arecoline 7.7 ± 0.6 pA, paired t-test, t _(17)_ = 0.078, *p* = 0.94, *n* = 18 cells/ 7 mice; Fig. 5a and b). In addition, the arecoline had no significant effect on the mIPSC (frequency: control 3.2 ± 0.7 Hz vs. arecoline 3.0 ± 0.75 Hz, paired t-test, t _(19)_ = 0.4427, *p* = 0.66; amplitude: control 13.5 ± 1.2 pA vs. arecoline 13.2 ± 1.5 pA, paired t-test, t _(19)_ = 0.23, *p* = 0.82, *n* = 20 cells/ 7 mice; Fig. 5c and d). These results indicate that the arecoline had no significant effect on the spontaneous synaptic transmission.

### The selective agonist inhibits both the evoked and spontaneous synaptic transmission in the ACC

**Fig. 6:**
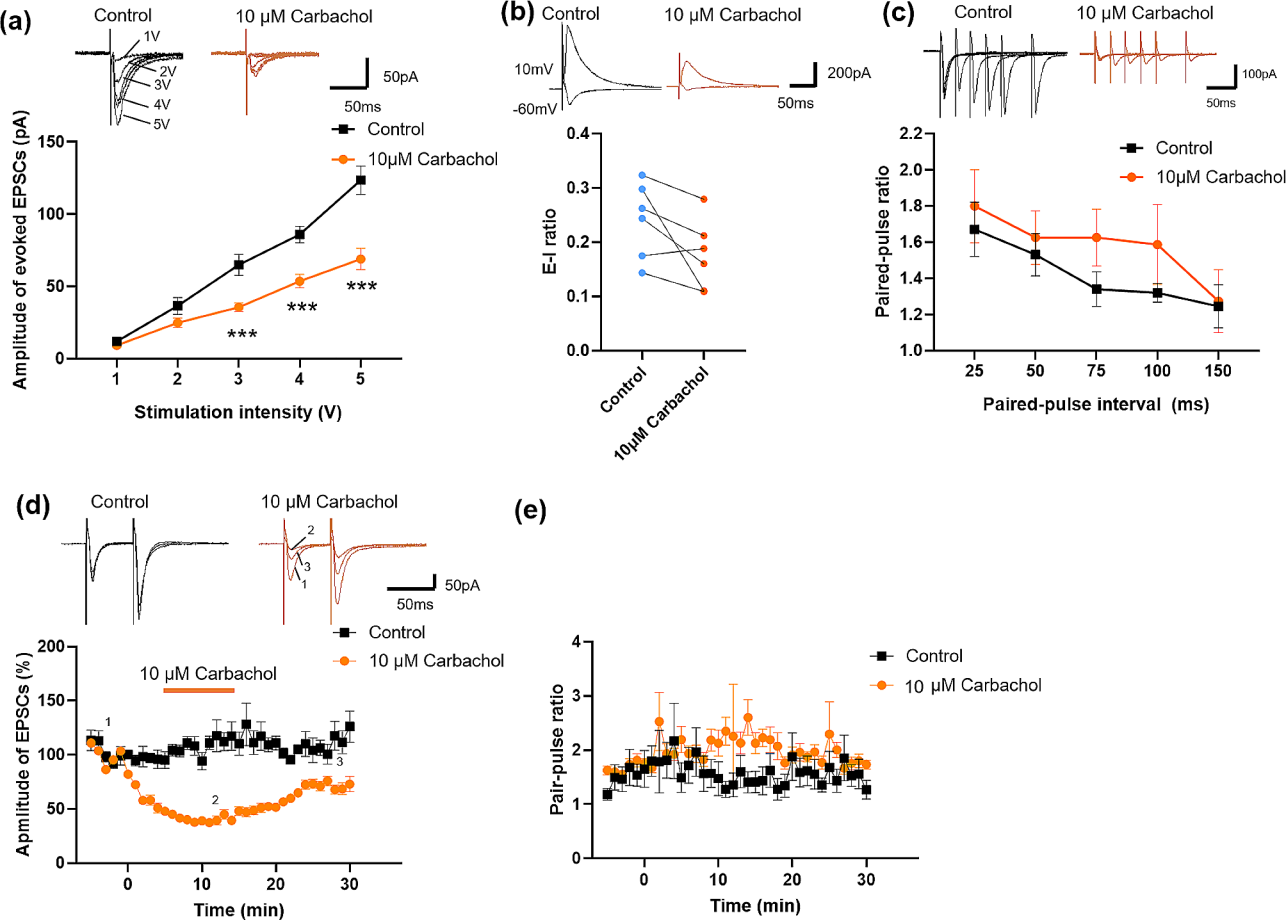
Carbachol decreased evoked synaptic transmission in layer II/III of the ACC. (**a**) Representative traces (top) and input-output curves (bottom) in ACC slices before and after the carbachol application. *n* = 6 neurons/3 mice. ***p < 0.001 compared with baseline control. (**b**) Top: representative traces of evoked excitatory and inhibitory responses recorded before and after the application of carbachol in the ACC. Bottom: the evoked E-I ratio before and after the application of carbachol. *n* = 6 neurons/3 mice. (**c**) Representative traces (top) and PPR before and after the carbachol application. *n* = 8 neurons/3 mice. (**d**) Representative traces (top) and plot of the time course of carbachol inhibiting the amplitude of paired pulse-induced EPSCs (bottom). The orange line indicates the time when the carbachol was perfused. (**e**) Time course of the PPR before, during, and after the application of carbachol. *n* = 16 cells/5 mice

Previous studies have reported that the arecoline is the agonist of the cholinergic receptor. We also found that the inhibitory effect of the arecoline in the ACC is dependent on the muscarinic receptors. Therefore, we applied another selective agonist of the muscarinic receptor, carbachol, to observe its effect on synaptic transmission. Similarly, the input-output curve was significantly shifted to the right after applying the carbachol (10 µM) compared with that in control group (*n* = 6 neurons/3 mice, two-way ANOVA, F _(1, 47)_ = 61.9 *p* < 0.0001, Fig. 6a). We found that both the excitatory and inhibitory evoked responses were inhibited after the application of carbachol, but the evoked E-I ratio had no significant change (control 0.24 ± 0.03 vs. carbachol: 0.18 ± 0.03, paired t-test, t _(5)_ = 2.3, *p* = 0.22, *n* = 6 cells/ 3 mice, Fig. 6b). Next, we recorded the PPR before and during the perfusion of carbachol. We found there was no significant change in PPR in ACC neurons compared with baseline (*n* = 8 neurons/3 mice, two-way ANOVA, F _(1, 70)_ = 2.932, *p* = 0.09, Fig. 6c). During the application of carbachol, the amplitude of EPSC decreased to 39.1 ± 3.8% of baseline (*n* = 16 cells/5 mice, Fig. 6d), while the PPR with the 50 ms interval had no significant change (Fig. 6e). These results suggest that the carbachol also inhibits the postsynaptic transmission in the ACC neurons.

**Fig. 7:**
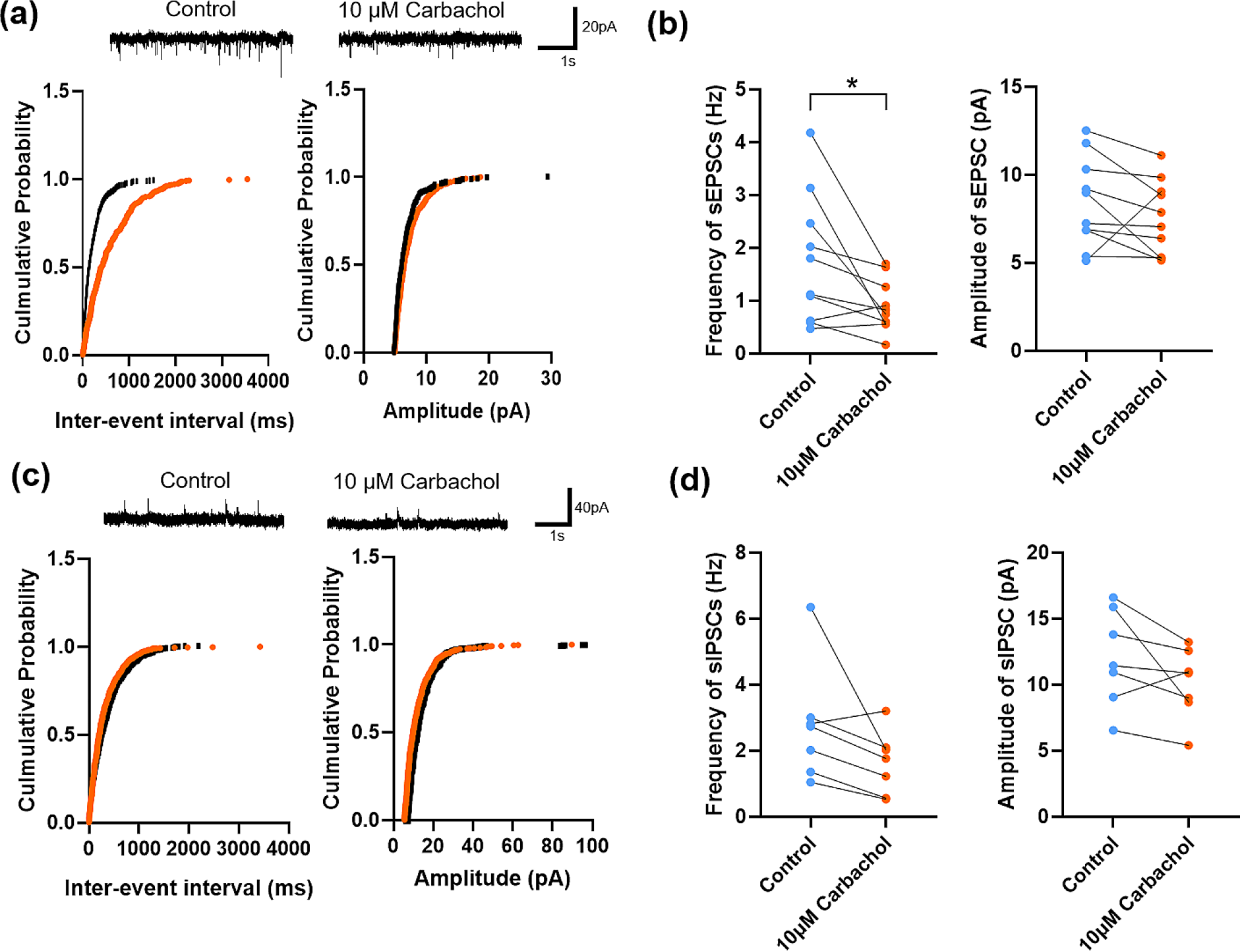
Changes of mEPSCs and mIPSCs in layer II/III of the ACC after the application of carbachol (**a**) Representative mEPSCs recorded in the ACC neuron in slices before (upper left) and after (upper right) the carbachol perfusion. Cumulative interevent interval (lower left) and amplitude (lower right) histograms of mEPSCs before (black square) and after (orange circle) the carbachol perfusion. (**b**) Summary plots of mEPSC data. Averaged values of mEPSC parameters: mean peak frequency (left) and amplitude (right). *n* = 10 cells/ 4 mice (**c**) Representative mIPSCs recorded in the ACC neuron in slices before (upper left) and after (upper right) the carbachol perfusion. Cumulative interevent interval (lower left) and amplitude (lower right) histograms of mIPSCs before (black square) and after (red circle) the carbachol perfusion. (**d**) Summary plots of mIPSC data. Averaged values of mIPSC parameters: mean peak frequency (left) and amplitude (right). *n* = 7 cells/ 3 mice

We also investigated the effect of carbachol on the mEPSCs and mIPSCs. Interestingly, the frequency of mEPSC decreased, while the amplitude did not change (frequency: control 1.7 ± 0.4 Hz vs. carbachol 0.9 ± 0.2 Hz, paired t-test, t _(9)_ = 2.628, *p* = 0.03; amplitude: control 8.4 ± 0.8 pA vs. carbachol 7.5 ± 0.7 pA, paired t-test, t _(9)_ = 1.291, *p* = 0.23, *n* = 10 cells/ 4 mice; Fig. 7a and b). The carbachol had no significant effect on the mIPSC (frequency: control 2.8 ± 0.7 Hz vs. carbachol 1.6 ± 0.4 Hz, paired t-test, t _(6)_ = 2.02, *p* = 0.09; amplitude: control 12.0 ± 1.4 pA vs. carbachol 10.1 ± 1.0 pA, paired t-test, t _(6)_ = 1.81, *p* = 0.12, *n* = 7 cells/ 3 mice; Fig. 7c and d). These results indicate that carbachol inhibits the spontaneous presynaptic release in neurons of the ACC.

## Discussion

The present study discovered the analgesic-, anxiolytic, or anti-depressive-like effect of the arecoline in mice of inflammatory pain, which is mediated by the inhibition of synaptic transmission in the ACC. This inhibition is mediated by the muscarinic receptors and is mainly found in the postsynaptic responses. Our study provides a different view of the pharmacological effect of areca nuts/ betel chewing on the central nervous system, although whether the arecoline could be used as a drug for the treatment of pain or pain-related anxiodepressive-like emotion remains to be discussed.

### The analgesic and anxiolytic effect of arecoline

Previous studies have reported the analgesic effect of arecoline via the muscarinic 1 and 2 receptors [15, 17, 18]. These reports investigated the analgesic effect after a single i.p application of arecoline in naive mice or described the mechanism at the spinal level. Although the anxiolytic effect of arecoline has been reported in humans [28], and one recent report investigating the anxiolytic effect in zebrafish [16], there is little research about its mechanism. In our results, by long-term applying the arecoline orally, we found that the arecoline caused analgesic, anxiolytic, and anti-depressive effects in normal mice and mice suffering inflammatory pain induced by CFA injection. This may be due to the inhibition of arecoline to the basal neural transmission, therefore, we then investigated the cortical mechanism of the analgesic and anxiolytic effect of arecoline in the ACC, a cortical area that is involved in pain and anxiety.

### Comparison of physiological vs. pathological conditions

There are a few analgesics that exert analgesic effects by inhibiting synaptic transmission. For example, lidocaine blocks sodium channels, gabapentin, and pregabalin block presynaptic calcium channels, and morphine inhibits the presynaptic release of substance P [29, 30]. Therefore, these analgesics inhibit basal synaptic transmission and may cause obvious side effects. In the present study, we found ACC transmission was inhibited by the application of arecoline under physiological conditions. At the behavioral level, we found that arecoline caused analgesic and anti-depressive effects under both physiological and pathological conditions. We compared it with a selective antagonist of adenylyl cyclase subtype 1 (AC1), NB001, which blocks the induction of cortical LTP without affecting basal neural transmission (Table 1). The NB001 alleviates the allodynic effect of chronic pain with few effects on the acute pain and anxiodepressive-like effect under physiological conditions [31]. As a comparison, in addition to the inhibition of synaptic transmission, the arecoline inhibits neurotransmission and has been proved to have a carcinogenetic effect. Loads of difficulties are required to be solved when using arecoline as a drug.

**Table 1:** Comparison of the effect of arecoline, carbachol, and NB001 on pain, anxiety, and depression under both physiological and pathological conditions

	Pain		Anxiety		Depression	
	Physiological	Pathological	Physiological	Pathological	Physiological	Pathological
Arecoline	✓	✓	×	✓	✓	✓
Carbachol	✓	✓	N/A	N/A	N/A	N/A
NB001	×	✓	×	✓	×	N/A

✓ effective; × noneffective; N/A not available

### Cholinergic transmission in the ACC

As an agonist of cholinergic receptors, the arecoline mainly activates the muscarinic M1 and M2 receptors in the central nervous system [28, 32]. Cholinergic projections play important roles in nociceptive modulation, and cumulative evidence has shown that activation of cholinergic projections in the central nervous system exerted analgesic effects [33, 34]. The muscarinic receptors are widely distributed in the ACC [35, 36]. In previous studies, pharmacological activation of the M1 receptor caused an analgesic effect in naïve animals by activating the GABA receptors [37, 38]. We did not find the activation of IPSCs in pyramid neurons of the ACC after the arecoline application. On the contrary, the evoked IPSC decreased with the arecoline as well as another muscarinic agonist carbachol, and the mIPSC had no significant change. This discrepancy may be due to the properties of agonists. Unlike McN-A-343, arecoline, as well as carbachol, are not selective agonists for the M1 receptor. The signaling pathway could be more sophisticated. However, activation of the M1 receptor in the ACC exerted an analgesic effect, which is similar to our reports. Since the neurons we recorded by whole-cell patch-clamp are pyramidal neurons, the decreased EPSC, IPSC, and increased E/I ratio indicate that the arecoline demonstrated inhibition on both the excitatory and inhibitory responses of pyramidal neurons but the inhibition on the inhibitory effect is stronger. This does not conflict with the net inhibition we measured by field potential, because the net inhibition may be caused by a bunch of neurons including the interneurons. For example, the activation of the muscarinic 1 receptor activates the parvalbumin (PV^+^) and somatostatin (SST^+^) interneurons [39–41]. Previous studies have reported that the activation of muscarinic receptors activates the GABA receptors [37, 38]. These may cause net inhibition. The signaling pathway of the arecoline remains to be further investigated.

Although both arecoline and carbachol are agonists for muscarinic receptors, in our study, we found that the inhibitory effects of the arecoline and the carbachol on the ACC neurons are slightly different. Previous studies reported that the arecoline is a partial agonist with peak effects of 30% of the maximum as obtained with carbachol [42]. In the rat cortices, administration of arecoline inhibited carbachol-stimulated phosphoinositide turnover. The most classical mechanism of the carbachol-stimulated phosphoinositide turnover is that activation of the muscarinic receptor would lead to a depolarization sufficient for the propagation of a spike [43]. This could open voltage-dependent Ca^2+^ channels and induce the release of a neurotransmitter that acts at a receptor directly coupled to the phosphoinositide response. Arecoline-stimulated desensitization is associated with sequestration of cell surface receptors without proteolytic degradation of internalized receptors [42]. Therefore, arecoline appears as a partial agonist in whole cells. This discrepancy may be the reason for the different inhibitory effects on the ACC neurons.

### Clinical indications of arecoline in acute pain, anxiety, and depression

Chronic pain has been proven to be comorbid with emotional disorders such as anxiety and depression. Analgesics that alleviate pain syndrome may also alleviate pain-related anxiety and depression. Although we found that arecoline caused analgesic and anxiolytic effects, and it has been used as a drug for the treatment of AD in its early years [44], cumulative evidence has been provided that the arecoline has strong side effects, since the arecoline has a wide spectrum of pharmacological effects in addition to the nervous system, modulating cardiovascular, digestive and endocrine systems as well as evoking a wide range of somatic effects, such as hypersalivation, hypotension, vertigo, miosis, tremor and bradycardia [1, 28]. Moreover, the well-known risk of arecoline use is its carcinogenic effect [32, 45, 46]. Therefore, we only interpret the pharmacologic effect of arecoline. To make the arecoline an internal medicine, the dependence and toxicity problems require to be dissolved.

## Data Availability

The datasets used and/or analyzed during the current study are available from the corresponding author on reasonable request.
